# In Vivo Discrimination of Iodine and Tantalum-Based Liquid Embolics After Intracranial or Spinal Embolization Using Photon-Counting Detector CT

**DOI:** 10.1007/s00062-025-01502-x

**Published:** 2025-02-06

**Authors:** Christoph Johannes Maurer, Ansgar Berlis, Franz Josef Stangl, Lars Behrens

**Affiliations:** Department of Diagnostic and Interventional Neuroradiology, University Hospital Augsburg, Stenglinstraße 2, 86156 Augsburg, Germany

**Keywords:** Photon-counting CT, Material decomposition, Iodine-based embolics, Tantalum-based embolics, Intracranial and spinal embolization

## Abstract

**Purpose:**

In vitro differentiation of iodine and tantalum-based liquid embolics post-embolization can be achieved using spectral computed tomography. This study evaluates the in vivo ability of clinical photon-counting computed tomography (PCD-CT) to distinguish these embolic agents in patients undergoing endovascular treatments for cerebrovascular and spinal pathologies.

**Methods:**

This retrospective study included 25 patients treated between April 2021 and March 2024, who underwent PCD-CT imaging post-embolization for intracranial arteriovenous malformations (AVM), dural arteriovenous fistulas (dAVF), spinal tumors, or middle meningeal artery (MMA) embolization for chronic subdural hematomas (cSDH). Imaging analysis involved iterative reconstruction, using conventional images (CI), iodine maps (IM), and virtual non-contrast (VNC) series. Two blinded neuroradiologists assessed the suppression quality of the embolic agents on a Likert scale.

**Results:**

Of the 25 patients, 22 underwent intracranial and 3 spinal embolizations. The differentiation between iodine and tantalum-based embolics achieved 92% accuracy for reader 1 and 88% for reader 2, with a Cohen’s kappa coefficient of 0.92 indicating high inter-reader agreement. Iodine-based agents were moderately suppressed, whereas tantalum-based agents exhibited superior suppression. Errors arose from mistaking suppressed platinum coils for tantalum-based embolics. Hemorrhage detection accuracy was high, with a Cohen’s kappa of 0.92.

**Conclusions:**

PCD-CT effectively differentiates between iodine- and tantalum-based embolics in vivo, demonstrating high diagnostic accuracy and inter-reader reliability. This capability facilitates improved post-procedural assessment and may enhance the management of endovascularly treated patients by reducing imaging artifacts and aiding in hemorrhage detection.

## Introduction

Liquid embolics are increasingly used for endovascular treatment of cerebral arteriovenous malformations (AVM) [1], dural arteriovenous fistulas (dAVF) [2] and more recently for the embolization of the middle meningeal artery (MMA) to prevent recurrence of chronic subdural hematomas (cSDH) [3]. To facilitate visualization of the liquid embolic during the embolization process, various radiopaque materials are employed, including tantalum, a transition metal and iodine, a halogen element. Liquid embolic agents that achieve radiopacity using iodine include cyanoacrylates (nBCAs and nHCAs) such as Histoacryl (B. Braun, Melsungen, Germany), Glubran 2 (GEM SRL, Viareggio, Italy), and Magic Glue (Balt, Montmorency, France). Cyanoacrylates themselves do not contain iodine; they are mixed before application with iodized oil (Lipiodol, Guerbet, Villepinte, France or formerly Ethiodol, Savage Laboratories, Melville, NY, USA), which provides visibility under fluoroscopy. More recently, the copolymer PHIL (MicroVention, Aliso Viejo, USA), which also uses iodine for visibility, has emerged as a promising option [4]. Embolic agents that typically use tantalum for visualization include the copolymers Onyx (Medtronic, Irvine, CA, USA), Squid (Balt, Montmorency, France) and Menox (Meril Life Sciences, Gujarat, India). The latest tantalum-based embolic agent is Lava (Black Swan Vascular Inc., Hayward, CA, USA), designed primarily for peripheral applications. Visualization of the embolic agent is of importance for the monitoring of the process and extent of embolization, as well as for the early detection of complications. One disadvantage of the extent of radiopacity necessary under fluoroscopy are artifacts, especially beam hardening artifacts in CT and CT-angiography after embolization [5]. These artifacts may complicate the assessment of brain tissue and surrounding vessels, thus potentially impacting diagnosis and therapy management. Conventional energy-integrating CT is prone to beam-hardening artifacts because it registers cumulative signal without distinguishing individual photon energies [6]. By contrast, photon-counting detector (PCD) technology uses semiconductor detectors that count and bin photons based on discrete energy thresholds, enabling more precise material decomposition, reduced noise, and inherently mitigating beam-hardening artifacts. Compared with dual-energy CT, which typically employs two spectra for material differentiation, PCD-CT offers in theory multiple energy bins for more refined spectral separation. This results in superior artifact reduction and enhanced visualization of high-attenuation material in a single CT scan at a fixed tube voltage [7, 8]. While both dual-energy CT (DE-CT) and photon-counting CT (PCD-CT) enable material decomposition, published comparisons indicate that this multi-bin technique confers improved material differentiation and reduced beam-hardening artifacts, particularly for high-attenuation embolic materials with a higher contrast-to-noise ratio than DE-CT at the same dose level [8–10]. This technology has the potential to facilitate material differentiation in any PCD-CT scan and opens new possibilities for K‑edge imaging, allowing the distinction of various materials based on their K‑edge characteristics within a single image acquisition [11] with possible application to post-interventional CT imaging in interventional neuroradiology. K‑edge imaging exploits the characteristic absorption edges (k-edges) of specific elements to distinguish them from surrounding tissues more accurately. By selecting narrow energy windows around these k‑edges, the system can enhance contrast for target materials, such as iodine or tantalum, facilitating enhanced material differentiation compared to conventional imaging methods. In vitro, the differentiation of tantalum and iodine using spectral K‑edge imaging has already been demonstrated [12]. Although multi-bin PCD-CTs are not yet clinically available, initial clinical experience with PCD technology has demonstrated significantly improved diagnostic capabilities and confirmed increased clinical value through the use of routinely reconstructed Iodine Maps (IM), which highlight the distribution and concentration of iodinated contrast agents to enhance vascular and lesion visualization, and Virtual Non-Contrast (VNC) images, which algorithmically remove iodinated contrast to simulate unenhanced scans and provide baseline tissue assessment [13]. Clinically, differentiating tantalum from iodine on photon-counting CT may allow more accurate assessment of incomplete AVF or AVM closure to guide subsequent therapies—particularly radiosurgery—and help distinguish hemorrhage from contrast medium extravasation. However, the primary objective of this study was to investigate the technical feasibility of in vivo post-procedural differentiation of tantalum and iodine-based liquid embolics using a clinical PCD-CT.

## Materials and Methods

### Study Design

This is a single-center retrospective observational study. The study was approved by the Institutional Review Board of the Ludwig-Maximilian-University of Munich (reference: 24-0267, date of approval 21/May/2024). The study was conducted in accordance with the ethical standards of the Declaration of Helsinki of 1964 and its subsequent amendments. Due to the retrospective design and the irreversible anonymization of patient data, the requirement for individual informed consent was waived.

### Patients

All consecutive patients treated between April 2021 and March 2024 who underwent postprocedural CT imaging on a novel PCD-CT (NAEOTOM Alpha; Siemens Healthineers, Erlangen, Germany) with a standard clinical protocol after embolization for cerebral or spinal dAVFs, AVMs, spinal tumors, or MMA for cSDH were included in the analysis.

### Scanning Protocol

All scans were performed on a photon-counting CT (PCD-CT) system in routine clinical practice. For cranial CTs, the protocol included an acquisition mode with spectral information display (QuantumPlus, Siemens Healthineers), a tube voltage of 120 kVp, automatic tube current modulation (Care DOSE 4D), a 0.5‑s rotation time, 0.55 pitch, and 0.4-mm collimation. Full-volume spectral series were reconstructed using a soft tissue kernel and a bone kernel (Hr68) with medium-level iterative reconstruction (QIR 2) and saved in an enhanced DICOM format (SPP, spectral post-processing) containing the full spectral information. The mean CTDI_vol for cranial CTs was about 43 mGy, depending on the extent of high-attenuation embolic material.

For spine CTs, a tube voltage of 20 kVp with automatic tube current modulation was used, with a 0.5‑s rotation time, 0.8 pitch, and 0.4-mm collimation. Full-volume spectral series were generated using a soft tissue kernel (Qr40) and a bone kernel (Qr68). The CTDI_vol ranged from 5.47 to 9.02 mGy, varying according to the anatomical region.

### Image Reconstruction and Analysis

Images were iteratively reconstructed on the scanner console using the Qr40f convolution kernel and a QIR level of 2. Spectral post-processing series were generated to preserve spectral image information for further analysis. Image analysis was then performed on a dedicated workstation (syngo.via, version VB80C; Siemens Healthineers, Erlangen, Germany) in a dual-energy workflow (virtual unenhanced application profile). Conventional images (CI), IM and VNC series were reconstructed.

### Image Evaluation

Two experienced diagnostic and endovascular neuroradiologists (CJM and LB) evaluated the reconstructed images using CI, IM, and VNC series. The neuroradiologists were blinded to the liquid embolic agents used, to potential hemorrhage and to possible complications, but not to the location of the embolization and the underlying pathology. All images were viewed at a standard window for brain parenchyma (width 40 HU, level 80 HU) or a soft tissue window for spinal evaluation (width 60 HU, level 400 HU) and a bone window (width 700 HU, level 3200 HU) using the full dynamic range. No additional cropping was applied, ensuring that the entire CT-value range was available for evaluation. They assessed whether the embolic agents were suppressed in the IM or the VNC series, and decided whether an iodine- or tantalum-based agent, or both, was used. They also rated the quality and extent of suppression of the embolic materials on a three-stage Likert scale as complete, moderate or incomplete and assessed whether additional hemorrhage was visible on the CT images. In this study, ‘suppression’ denotes the reduction or removal of the visible signal from a specific material (iodine or tantalum) in the VNC or IM images, achieved through material decomposition algorithms during post-processing [6]. The ground truth for the type of embolic agents used was established by the embolization protocol, while the presence of hemorrhage was determined by the clinical course and follow-up imaging on conventional EID-CT.

### Statistics

All statistical analyses were conducted using Python (version 3.9.7). Descriptive statistics were computed to summarize the distribution and central tendencies of the ratings provided by each reader. Cohen’s kappa coefficient was utilized to quantify the inter-reader agreement. The accuracy of each reader’s assessments compared to the ground truth was assessed using the Overall Accuracy, Precision, Recall, and the F1-score.

## Results

### Patients’ Characteristics and Embolic Agents

A total of 25 patients were included in the analysis. Of these, 22 had received an intracranial embolization, while 3 had undergone a spinal embolization. Of the 22 patients who had undergone an intracranial embolization, 8 had a cerebral AVM, 9 had a dAVF, and 5 had a cSDH. One patient had an AVM in the cervical spine, one patient had a post-surgical hemorrhage from a lumbar segment artery, which was treated with embolization, and one patient was embolized due to a metastasis of a renal cell carcinoma before surgery. A single agent was used in 19 cases, Menox in one case, Magic Glue in six cases, and PHIL in 12 cases. In six cases, a combination of embolic agents was employed. In four of these cases, a combination of iodine- and tantalum-based agents was utilized. The detailed characteristics are summarized in Table 1.

**Table 1:** Patients’ characteristics, t = tantalum-based, i = iodine-based

Characteristics	*N* = 23
**Age Median (IQR)**	62 (36, 68.5)
**Sex, *n* (%)**	
Male	19 (76%)
Female	6 (24%)
**Diagnosis, *n* (%)**	
Unruptured AVM	5 (20%)
Unruptured dAVF	8 (32%)
Ruptured AVM	4 (16%)
Ruptured dAVF	1 (4%)
cSDH	5 (20%)
Pre-op tumor embolization	1 (4%)
Intraoperative vessel perforation	1 (4%)
**Location, *n* (%)**	
Intracranial	22 (88%)
Spinal	3 (12%)
**Embolic agents, *n***	
Onyx (t)	1
Menox (t)	2
Squid (t)	2
Lava (t)	2
Magic Glue (i)	10
PHIL (i)	16

### Accuracy for Discrimination

The overall accuracy in differentiating iodine-based from tantalum-based embolic agents was 92% for reader 1 and 88% for reader 2. The Cohen’s kappa coefficient of 0.92 indicated highly concordant readings between the two readers. Detailed metrics are provided in Table 2.

**Table 2:** Reader accuracy for discrimination iodine vs. tantalum [%]

	Reader 1	Reader 2
*Overall Accuracy*	92	88
*Precision*	95	93
*Recall*	92	88
*F1-score*	93	89

### Quality of Suppression

**Fig. 1:**
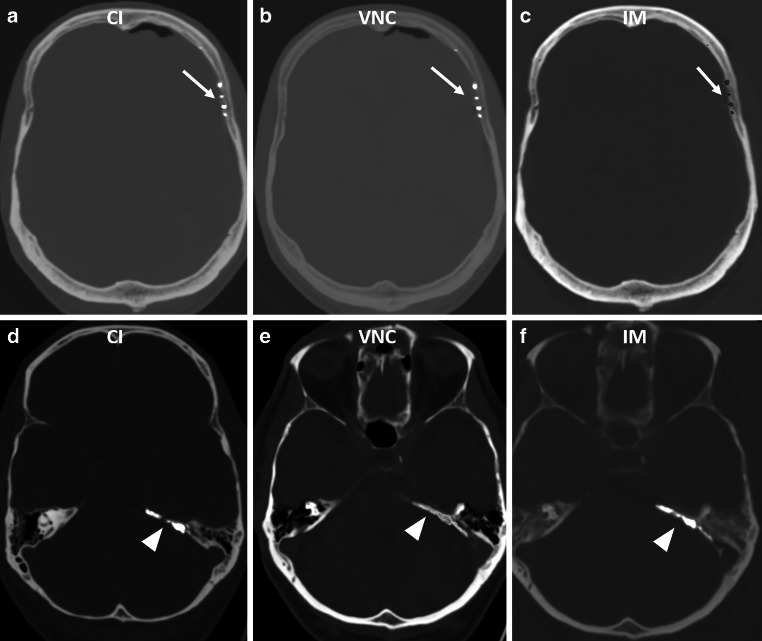
**a** to **c** Left MMA embolization with Menox (arrows) with suppression of the embolic agent in the iodine maps. Window settings: W: 200 HU, L: 1500 HU **d** to **f** Embolization of a tentorial fistula with PHIL (arrowheads). Note the incomplete suppression of the embolic agent on the VNC series. Window settings: *W* 500 HU, L : 1500 HU *CI* conventional imaging, *VNC* virtual non-contrast. *IM* iodine map

**Fig. 2:**
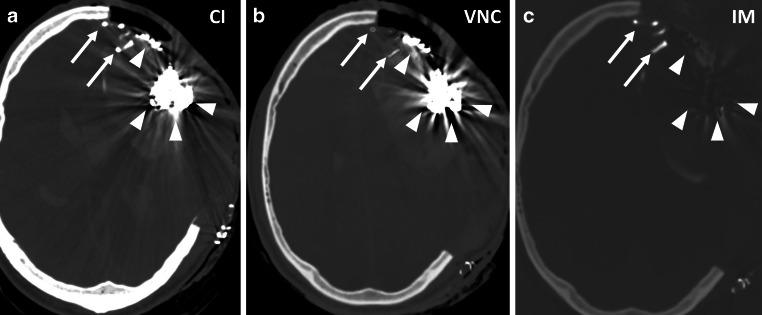
**a** to **c** Embolization of a left frontal AVM withLava (arrows) and PHIL (arrowheads). Lava as a tantalum-based embolic agent is suppressed in the IM, PHIL, as an Iodine based agent is partially suppressed in the VNC map. Window settings: W: 200 HU, L: 1500 HU. *CI* conventional imaging, *VNC* virtual non-contrast, *IM* iodine map

Reader 1 rated the iodine suppression with a median [IQR] of 2 [1–2], while Reader 2 rated it with a median [IQR] of 3 [1.5–3]. The agreement between the two readers was only moderate, with a Cohen’s kappa value of 0.40. Tantalum suppression was evaluated as 1 [1–1] by both readers. However, an interrater agreement could not be calculated reasonably due to the small number of cases, with only six patients receiving tantalum-based embolic agents and in five of these cases an iodine-based embolic agent was used as well. Examples of Iodine and tantalum-based embolic agents in CI, IM and VNC series are provide in Fig. 1. An example of a patient treated with both, Iodine and tantalum-based embolic agents is presented in Fig. 2.

### Identification of Hemorrhage

**Fig. 3:**
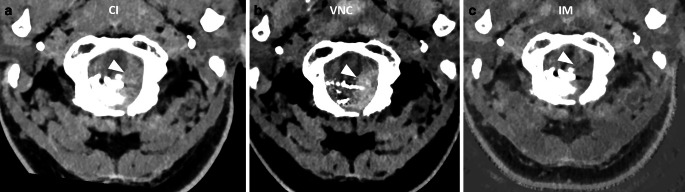
**a** to **c** Embolization of a dAVF at the foramen magnum with PHIL. The SAH (arrowheads) is best visualized on the VNC with suppression of a large part of the cast. Window settings manually optimized for contrast: **a** W: 75 HU, L: 150 HU **b** W: 40 HU, L: 80 HU **c** W: 80 HU, L : 350 HU. *CI* conventional imaging, *VNC* virtual non-contrast, *IM* iodine map

Hemorrhage was identified in 16 cases, including intraventricular hemorrhage, subarachnoid hemorrhage (SAH), parenchymal hemorrhage, and subdural hemorrhage. Reader 1 achieved an overall accuracy of 92%, while Reader 2 demonstrated an accuracy of 96% with a Cohen’s kappa of 0.92, indicating a very high level of agreement between the two readers. An example of an iodine-based liquid embolic next to a SAH is presented in Fig. 3.

### Interpretation Errors

**Fig. 4:**
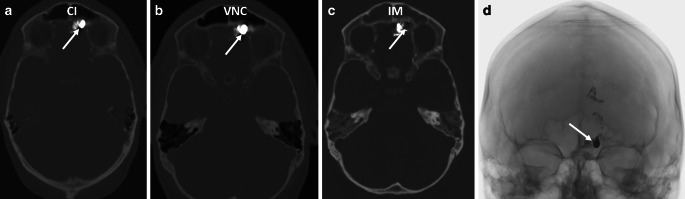
**a** to **c** Embolization of a fronto-basal dAVF with PHIL and coils. Note the suppression of the coils (arrows) in the iodine map. W: 200 HU, L: 1500 HU. **d** Single shot image during intervention, the arrow points to the coils within the PHIL cast. *CI* conventional imaging, *VNC* virtual non-contrast, *IM* iodine map

Both readers evaluated two cases of intracranial dAVF and incorrectly identified them as containing both iodine and tantalum-based liquid embolics, while only PHIL was actually used in both cases. Platinum coils were utilized in these patients and these coils were suppressed in the IM and were thus mistaken for tantalum-based embolics. Specifically, one case involved a tentorial fistula treated for a flow-related aneurysm, and the other case involved a fronto-basal fistula to reduce reflux in a transvenous approach. All other cases were treated without platinum coils. The corresponding images for the second case are shown in Fig. 4.

## Discussion

Our data demonstrate that iodine-based and non-iodine-based embolic agents can be reliably distinguished on routinely acquired IM and VNC images on PCD-CTs following endovascular therapy of vascular pathologies of the brain and spine. To our knowledge, this is the first study to provide in vivo evidence that intracranial discrimination of iodine and tantalum-based embolic agents is possible with a very low inter-reader variability. This information may be useful in evaluating the distribution of different embolic agents, given the increasing availability of iodine-based and non-iodine-based embolics [4, 14]. Although differentiating between iodine- and tantalum-based agents is usually not necessary—given that the chosen agent is typically documented—this distinction can be useful when tantalum-based materials are employed, particularly to differentiate the embolic agent from postinterventional iodine-based contrast extravasation. Moreover, in partially treated arteriovenous malformations or fistulas, precisely identifying which territories remain perfused by contrast versus those occluded by tantalum-based embolics can inform further endovascular interventions or guide radiosurgical planning. In large fistulas or AVMs where multiple agents are used, localizing each agent may help clarify the lesion’s anatomy. Furthermore, imaging artifacts can be effectively reduced to allow for more accurate evaluation of potential post-procedural complications such as hemorrhage or ischemia [15]. In multiphase treatment or long-term follow-up, discrimination of different embolic agents may also be helpful in evaluating treatment success and planning further interventions. Identifying postinterventional novel embolic agents, such as _iht_Obtura—an iodine-based agent whose radiopacity diminishes over time—may be clinically relevant [16]. PCD-CT’s high spectral resolution can confirm the agent’s distribution soon after deployment, before opacification fades, thereby influencing subsequent treatment decisions. Elements with higher atomic numbers, such as tantalum, have been identified as potential new X‑ray contrast agents for PCD-CTs [17–19]. However, tantalum-specific maps, similar to the iodine maps used in dual-energy technology and PCD-CT or tantalum-specific K‑edge imaging on experimental scanners, are not yet available on clinical scanners. This is the reason, why the coils in two of our cases with dAVF were suppressed within the PHIL cast on IM in our data and were therefore mistakenly considered to be tantalum-based embolic agents. As technology advances, K‑edge imaging for different elements may become a routinely used method to detect tantalum-based materials or other contrast agents than iodine and reduce subsequent artifacts caused by these elements. Particularly in cases of AVMs or fistulas where large casts can been created during endovascular embolization, this could provide a valuable improvement in image quality in the future.

In our study, the suppression of tantalum was found to be superior to that of iodine-based liquid embolic agents. In PHIL, iodine is chemically bound to the copolymer to provide radiopacity for fluoroscopic visualization. Unlike Onyx or Squid, PHIL does not require shaking prior to delivery and produces fewer artifacts on CT [20]. However, the reasons for the incomplete suppression on VNC maps in compared to tantalum suppression on IM are unclear. This discrepancy may stem from the different physical properties, particularly the higher atomic number of tantalum and their concentrations, which might influence suppression on PCD-CT depending on the energy levels used. The difference in distribution and bonding is unlikely to be a significant factor. Advanced post-processing techniques may help resolve this issue in the future.

In our study, accuracy and inter-reader reliability were high, which is relevant for detecting hemorrhage as a potential complication of endovascular therapy for intracranial lesions. However, direct comparisons to energy-integrating detector CT were not performed, and further investigations are needed to confirm these results. The ability to distinguish between iodine and hemorrhage, particularly after endovascular thrombectomy in ischemic strokes, has been demonstrated using dual-energy CTs [7, 21, 22]. Suppressing iodine or tantalum and reducing beam-hardening artifacts from these agents enhances the visualization of hemorrhage or contrast media extravasation after embolization, aiding in risk assessment post-therapy [23]. In our patients, accompanying hemorrhage was reliably detected in nearly all cases, especially where adequate cast suppression was achieved, even in challenging locations such as the cranio-cervical junction (Fig. 2). The potential of artefact reduction with PCD-CT has already been demonstrated [24, 25]. These techniques may resolve the challenge of achieving excellent visualization of embolic agents during fluoroscopy while minimizing artifacts in post-intervention CT imaging.

Our study has inherent limitations, including its retrospective design, a relatively small sample size due to the rarity of the diseases, and the heterogeneity of the embolic agents, which collectively affect the robustness of our data. Additionally, the evaluation was performed by two experienced neuroradiologists, which may limit its applicability to less experienced readers. Nevertheless, despite these limitations, our study demonstrates excellent accuracy in differentiating between various embolic agents and detecting hemorrhage, suggesting that PCD-CT has significant potential for use in post-interventional imaging.

## Conclusion

In vivo PCD-CT enables the discrimination between iodine- and non-iodine-based liquid embolics following intracranial and spinal embolization. Furthermore, PCD-CT allows for the detection of associated hemorrhage despite the presence of radiopaque liquid embolics. Further comparisons with conventional CT are needed to assess whether PCD-CT provides superior diagnostic capabilities and clinical benefits for these specific imaging challenges.

## Abbreviations

AVM: Arteriovenous Malformation
dAVF: Dural Arteriovenous Fistula
DE-CT: Dual Energy CT
MMA: Middle Meningeal Artery
cSDH: Chronic Subdural Hematoma
PCD-CT: Photon-Counting Detector Computed Tomography
CI: Conventional Imaging
IM: Iodine Maps
VNC: Virtual Non-Contrast
PHIL: Precipitating Hydrophobic Injectable Liquid
SAH: Subarachnoid Hemorrhage
nBCAs: n‑Butyl Cyanoacrylates
nHCAs: n‑Hexyl Cyanoacrylates
